# Response to comments on “Impact of climate change on dentistry and oral health: a scoping review”

**DOI:** 10.1038/s41405-025-00396-8

**Published:** 2026-01-05

**Authors:** Harsh Priya, Upendra Singh Bhadauria, Bharathi M. Purohit, Nicolas Giraudeau, Mansi Atri

**Affiliations:** 1 Centre for Dental Education and Research, All India Institute of Medical Sciences New Delhi, New Delhi, India; 2 Vital Strategies, New Delhi, India; 3 Université de Montpellier, Montpellier, France; 4 ESIC Dental College and Hospital, New Delhi, India

**Keywords:** Dental public health, Oral cancer

## Dear Editor,

We thank the correspondents for their thoughtful engagement with our article, “Impact of climate change on dentistry and oral health: A scoping review” [1]. We appreciate their recognition of our effort to synthesise a rapidly evolving interdisciplinary field and welcome the opportunity to clarify and expand upon the points raised.

## Reference accuracy

We appreciate the correspondent’s observation regarding the citation of the article by Sri Lakshmi Ajit. The source in question was cited as “Ajit SL. Effects of Climate Changes on Oral Health. Oral Health Case Rep. 2021;7:32.” The published version of the journal included the line “How to cite this article: Sri Lakshmi Ajit, Effects of Climate Changes on Oral Health. Oral Health Case Rep 6(2020) 8:21,” which we followed according to the citation format displayed in the article itself at the time of data extraction. The apparent discrepancy arises because the publisher’s metadata and indexing services list the article under the 2021 volume, whereas the in-text “How to cite” note within the PDF retains the earlier 2020 volume notation. To maintain consistency with the publisher’s online database, we cited the 2021 entry. The duplication noted in our references occurred during reference management import, and we acknowledge that both entries referred to the same author’s works. We thank the correspondents for identifying this, which helps enhance the bibliographic precision of our review [2].

## Inclusion of the Grose et al. [3] study

Our scoping review adopted an intentionally broad inclusion framework, consistent with the Joanna Briggs Institute (JBI) methodology, to capture both direct and indirect intersections between climate change and dentistry. The study by Grose et al. explored the knowledge and attitudes of dental staff toward sustainability and resource management, explicitly including “knowledge and awareness of climate change” as part of its qualitative interview framework. This study thus examined how dental professionals perceive and respond to climate-related issues within the context of everyday clinical practice.

Although the paper’s primary focus was not on oral health outcomes, it addressed an essential dimension of professional adaptation to environmental pressures. Moreover, the In Brief section of the same publication, an official editorial synopsis, highlighted that “climate change will negatively affect health service provision” and that “resources may become scarce due to extreme weather events.” These statements were relevant to our review’s thematic mapping of literature linking climate stressors to healthcare resource vulnerability, including dentistry.

Given that the objective of a scoping review is to map the range and nature of existing evidence rather than to restrict inclusion to studies demonstrating direct causality, the inclusion of Grose et al. [3] was methodologically justified. The study contributed to the thematic category of “sustainability in dental service delivery and resource use under environmental stress.” We agree, however, that distinguishing between direct epidemiological studies and contextual or behavioural research is essential for clarity in evidence synthesis, and we appreciate the opportunity to elaborate on this rationale.

## Epidemiological evidence

We concur that studies examining air pollution and ultraviolet radiation exposure offer valuable insights into the environmental determinants of oral disease. The cited examples from Taiwan and China further support our central argument, while empirical evidence is emerging, it remains dispersed across disciplines and rarely integrated within a unified climate oral health framework. Our conclusion that further structured epidemiological studies explicitly linking climate-related exposures with oral health outcomes are needed remains consistent with these findings.

## Recommendations on education and sustainable practice

We greatly appreciate the correspondents’ emphasis on embedding sustainability within dental education and clinical guidance. Although briefly mentioned in our “future directions,” we agree that incorporating climate-conscious principles into dental curricula, professional regulation, and practice is vital for strengthening environmental responsibility and resilience within the dental workforce. This represents an important avenue for future policy and research development.

## Conclusion

We sincerely thank the correspondents for their constructive observations, which enrich scholarly dialogue on the intersection of climate change and oral health. Such exchanges advance our shared goal of promoting evidence-based, environmentally responsible dentistry and public-health practice. We hope this discussion encourages continued interdisciplinary collaboration and integration of sustainability within dental research, education, and care delivery.
